# Should glucose screening be repeated for large-for-gestational-age fetuses in the third trimester of non-diabetic pregnancies? A scoping review

**DOI:** 10.61622/rbgo/2026rbgo76

**Published:** 2026-10-30

**Authors:** Caio Ribeiro Vieira Leal, Mário Dias Corrêa

**Affiliations:** 1 Universidade Federal de Minas Gerais Faculdade de Medicina Belo Horizonte MG Brazil Faculdade de Medicina, Universidade Federal de Minas Gerais, Belo Horizonte, MG, Brazil.; 2 Universidade Federal de Minas Gerais Hospital das Clínicas Belo Horizonte MG Brazil Hospital das Clínicas, Universidade Federal de Minas Gerais, Belo Horizonte, MG, Brazil.

**Keywords:** Pregnancy, Pregnancy trimester, third, Diabetes, gestational, Obesity, maternal, Hyperglycemia, Large-for-gestational age, Fetal macrosomia, Shoulder dystocia, Labour induced, Intensive care units, neonatal

## Abstract

**Objective:**

To map and synthesize global evidence regarding the clinical utility, diagnostic yield, and perinatal impact of repeating third-trimester glucose screening in previously screen-negative, non-diabetic pregnancies complicated by ultrasound-suspected large-for-gestational-age (LGA) fetuses or macrosomia, evaluating whether late dysglycemia explains accelerated growth.

**Methods:**

Following defined criteria for singleton pregnancies with normal mid-trimester testing and late-onset suspected fetal overgrowth, a systematic search was performed in June 2026 across PubMed, Embase, and SciELO for peer-reviewed articles in English, Portuguese, and Spanish. Two independent reviewers handled selection, rigorous critical appraisal, and data extraction for qualitative narrative synthesis.

**Results:**

Fifteen observational studies met inclusion criteria (nine retrospective cohorts, four prospective cohorts, two case-controls). Diagnostic yield for late-onset gestational diabetes mellitus (GDM) ranged from 2.9% to 24.6%. Diagnosing late GDM consistently changed clinical management, driving higher rates of labor induction and cesarean deliveries. Active standard-of-care maternal interventions initiated after retesting effectively curtailed excessive growth trajectories, producing no statistically significant differences in acute neonatal morbidities, such as shoulder dystocia or neonatal intensive care unit admissions, compared to non-diabetic controls.

**Conclusion:**

Indiscriminate repeat third-trimester glucose testing for isolated LGA fetuses cannot be routinely recommended due to high methodological heterogeneity and no randomized controlled trials (RCTs). Fetal overgrowth remains predominantly multifactorial, mediated by maternal obesity and dyslipidemia. Clinical management warrants careful individualization, and robust interventional RCTs are needed.

**Open Science Framework:**

https://doi.org/10.17605/OSF.IO/TFH7A

## Introduction

Suspected macrosomia and large-for-gestational-age (LGA) fetuses represent a growing challenge in modern obstetrics. These conditions have distinct definitions: a fetus is typically considered LGA when its estimated fetal weight (EFW) or abdominal circumference (AC) is above the 90th percentile for its gestational age, though other thresholds like the 95th or the 97th percentiles, +2 standard deviations, or +2 Z-score deviations are also used.^(1)^ In contrast, macrosomia at term usually refers to an absolute birth weight above a fixed cut-off of 4000 g or 4500 g.^(1)^ The prevalence of LGA infants and macrosomia varies widely in the literature, with LGA reported between 5% and 18%, and macrosomia between 1% and 9% globally,^(2–5)^ displaying a steady upward trend over recent decades driven by shifting demographic risk factors.^(2,6,7)^ These clinical entities are inherently associated with a substantial increase in adverse perinatal and maternal outcomes, including shoulder dystocia, birth trauma, increased rates of emergency cesarean deliveries, perinatal mortality, and short- and long-term metabolic sequelae for both mother and offspring, especially if associated with maternal diabetes.^(3,8–11)^

While multiple maternal and environmental factors contribute to accelerated fetal growth, gestational diabetes mellitus (GDM) remains one of the most clinically significant condition driving this pathophysiological pathway.^(12,13)^ International professional society guidelines heavily emphasize timely GDM screening during prenatal care, typically recommended during the first and/or second trimesters of gestation to mitigate these risks.^(14–16)^ Currently, screening and diagnosis of GDM between 24 and 28 weeks of gestation vary among major medical organizations, and local protocols may adopt different strategies. The 2024 American Diabetes Association (ADA) guidelines recommend a single-step approach using the 75-g oral glucose tolerance test (OGTT).^(17)^ Under this one-step criteria, GDM is diagnosed if at least one blood glucose value meets or exceeds the following cutoffs: fasting ≥ 92 mg/dL (5.1 mmol/L), 1-h ≥ 180 mg/dL (10.0 mmol/L), or 2-h ≥ 153 mg/dL (8.5 mmol/L). Although other diagnostic criteria may also be utilized in clinical practice,^(18)^ the American College of Obstetricians and Gynecologists (ACOG) guidelines recommend a two-step approach.^(19)^ The screening begins with a 50-g glucose challenge test (GCT), followed by a 3-h 100-g OGTT if the initial value exceeds institutional cut-offs, commonly 130–140 mg/dL (7.2–7.8 mmol/L). Under ACOG criteria, GDM is diagnosed when two or more values in the 100-g OGTT exceed the Carpenter and Coustan cutoffs: fasting ≥ 95 mg/dL (5.3 mmol/L), 1-h ≥ 180 mg/dL (10.0 mmol/L), 2-h ≥ 155 mg/dL (8.6 mmol/L), and 3-h ≥ 140 mg/dL (7.8 mmol/L).

It is crucial to recognize that not every LGA fetus is driven by maternal hyperglycemia. For instance, in a 5-year case-control study conducted at our institution, only 30% of macrosomic newborns were born to mothers with diabetes.^(9)^ Several other maternal and environmental factors are strongly associated with fetal macrosomia, including excessive gestational weight gain, pre-pregnancy maternal obesity, multiparity, advanced maternal age, and constitutional genetics.^(20–23)^ Despite this multifactorial etiology, GDM remains the most heavily scrutinized condition among health professionals in patients with a LGA fetus. Consequently, a substantial proportion of non-diabetic pregnancies presenting with LGA fetuses are still viewed almost exclusively through the lens of glucose intolerance.

This disproportionate clinical focus creates a dilemma in managing patients with normal initial screening who present with an LGA fetus in the third trimester. Whether these patients should undergo repeat glucose testing remains highly controversial. Given the absence of an international consensus on the clinical utility and timing of retesting in this specific population, current practices vary widely. Therefore, the aim of this narrative review is to synthesize the available literature regarding third-trimester repeat glucose testing for LGA fetuses, evaluating its diagnostic yield, impact on maternal and neonatal outcomes, and overall necessity in modern obstetric management.

## Methods

### Protocol and registration

This scoping review was conducted in accordance with the Joanna Briggs Institute (JBI) methodology for scoping reviews and is reported following the Preferred Reporting Items for Systematic Reviews and Meta-Analyses extension for Scoping Reviews (PRISMA-ScR) guidelines. The study protocol was prospectively registered on the Open Science Framework (OSF) under the identifier tfh7a (DOI: https://doi.org/10.17605/OSF.IO/TFH7A).

### Eligibility criteria

The inclusion and exclusion criteria were established a priori based on the PCC (Population, Concept, Context) framework:

- Population: Pregnancies with an initially normal GDM screening that developed LGA fetuses or suspected macrosomia in the third trimester. Studies focusing exclusively on pre-existing diabetes or early-diagnosed GDM cohorts were excluded.
- Concept: The utilization of third-trimester repeat glucose screening or alternative glycemic testing. The focus encompasses the diagnostic yield of repeat testing, its clinical utility, and its impact on maternal and neonatal outcomes.
- Context: Global obstetric practices, including studies evaluating clinical variations, management dilemmas, and official protocols from major international obstetric societies. Eligible study designs included peer-reviewed randomized controlled trials, prospective and retrospective cohort studies, systematic reviews, and official society guidelines. Searches were restricted to articles published in English, Portuguese, and Spanish. Case reports, editorials, non-peer-reviewed preprints, and studies lacking isolated extractable data on third-trimester re-screening outcomes were excluded.

### Information sources and search strategy

A comprehensive systematic search was executed across three major electronic databases: PubMed/MEDLINE, Embase, and SciELO, covering literature from inception up to June 2026. The search strategy utilized a tailored matrix combining controlled vocabulary (MeSH terms) and free-text keywords with Boolean operators: ("large-for-gestational-age" OR "LGA" OR "fetal macrosomia") AND ("third trimester" OR "third-trimester") AND ("screening" OR "re-screening" OR "repeat testing" OR "oral glucose tolerance test" OR "OGTT") AND ("gestational diabetes mellitus" OR "GDM"). Detailed full search strings are available in supplementary material - table 1S.

### Selection of sources of evidence

Following the execution of the search strategy and the removal of duplicates, the selection process was conducted in two stages. First, titles and abstracts were independently screened by the reviewing team against the predefined eligibility criteria. Subsequently, the full texts of potentially relevant articles were retrieved and assessed. Any discrepancies regarding study relevance between reviewers were resolved through consensus or by consultation with a senior investigator to minimize selection bias.

### Data extraction

Data charting was conducted using a standardized extraction format (Supplementary Material – Table 2S). Extracted variables included study characteristics (author, year, design, country), cohort demographics (sample size, maternal pre-pregnancy BMI, gestational weight gain, parity, and LGA/macrosomia diagnostic thresholds), and screening parameters (gestational age at repeat testing, type of diagnostic test, and conversion rate to GDM). Furthermore, clinical and perinatal outcomes (e.g., shoulder dystocia, birth trauma, emergency cesarean deliveries, and NICU admissions) were recorded.

### Data analysis and synthesis

Given the design of a scoping review, a formal quantitative meta-analysis was not performed. Instead, a qualitative narrative synthesis was conducted to map the current landscape of the literature. Data were synthesized using descriptive summary tables and thematic categorizations to delineate clinical yields, variations in obstetric care, and contradictions within existing international guidelines regarding third-trimester re-screening protocols.

## Results

### Study selection

The systematic literature search yielded a total of 494 records across the three electronic databases searched. After the removal of 176 duplicate records, 318 unique records underwent title and abstract screening. Of these, 266 records were excluded for not meeting the inclusion criteria. Among the 52 remaining records, 7 reports could not be retrieved, even after contact with the authors. The remaining 45 articles were retrieved for full-text assessment, from which 30 were subsequently excluded. Ultimately, 15 studies met all eligibility criteria and were included in this scoping review.^(24–38)^ The complete study selection process is detailed in the PRISMA flowchart (Figure 1).

**Figure 1:**
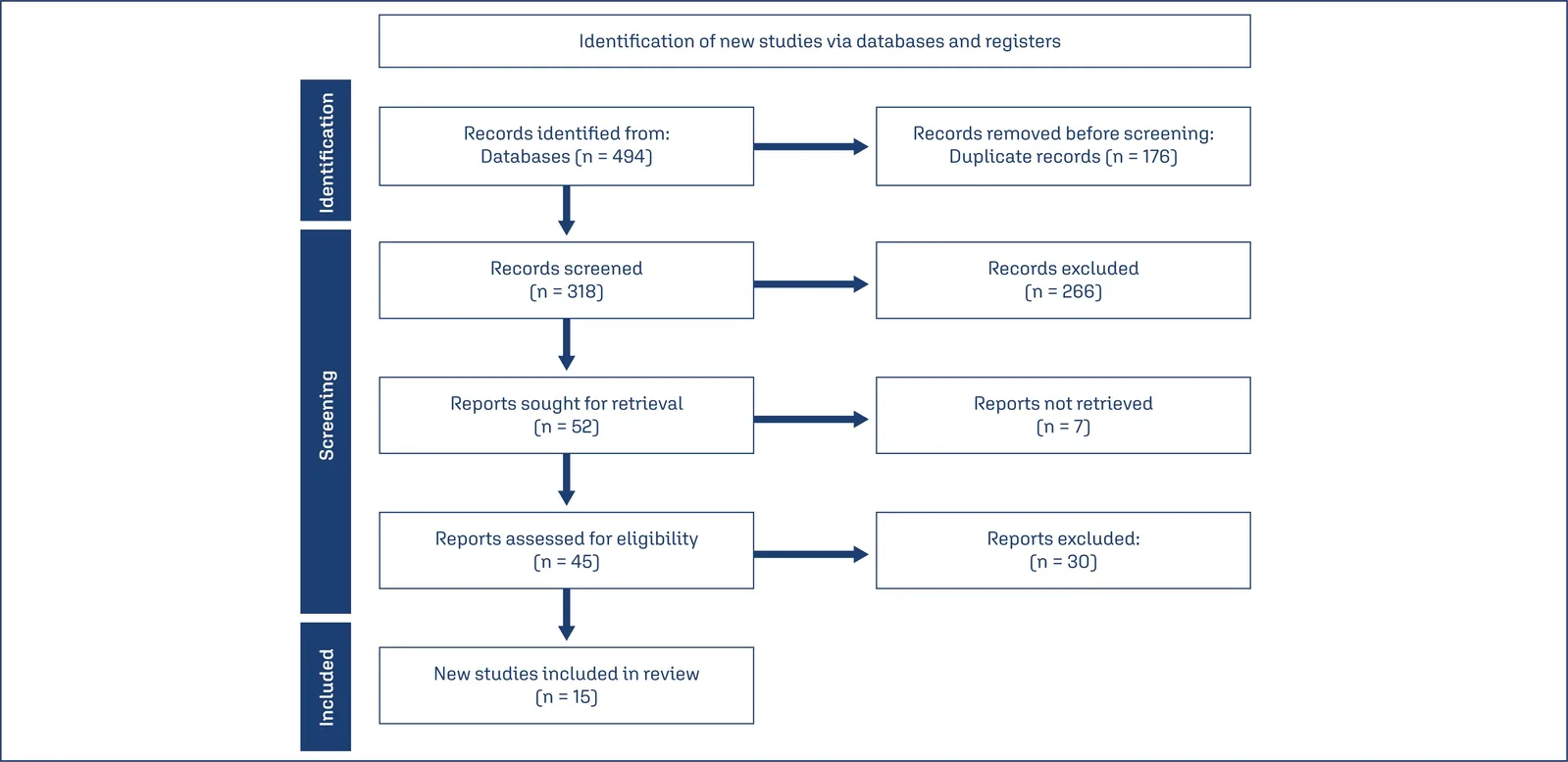
Screening of studies for inclusion in this scoping review

### Study characteristics

The clinical and methodological characteristics of the 15 included studies are summarized in chart 1.

**Chart 1:** Baseline characteristics and diagnostic methodologies of the included studies

Study (Author, Year)	Country	Study design, Publication type	GDM rescreening criteria	Diagnostic method and GDM criteria	Population characteristics and sample size
Abu Shqara, 2022^(24)^	Israel	Retrospective cohort, Full-length article	Suspected LGA (EFW > 90th percentile), polyhydramnios, pathological GCT at 24-28 weeks not followed by an OGTT, or other GDM predisposing factors (BMI > 30 kg/m², family history of DM, or personal history of GDM)	100-g OGTT; Carpenter-Coustan criteria*	372 women who underwent OGTT after 29 weeks, of whom 126 had LGA fetuses
Barak, 2021^(25)^	Israel	Retrospective cohort, Full-length article	Suspected macrosomia (EFW > 90th percentile) and/or polyhydramnios (not specified)	100-g OGTT; Carpenter-Coustan criteria*	272 women who underwent OGTT at term (37–42 weeks), of whom 147 had suspected isolated macrosomia
Cauldwell, 2022^(26)^	UK	Retrospective cohort, Full-length article	2+ glycosuria on a single urine dipstick, repeated minor glycosuria (1+ or 2+ on two separate occasions), suspected macrosomia (EFW > 95th percentile), or fetal AC > 95th percentile	Home capillary blood glucose monitoring for 1 week (4 times a day); Late-onset GDM was diagnosed if there were ≥ 3 abnormal readings within 7 days (fasting ≥ 5.3 mmol/L [95 mg/dL] or 1-hour postprandial ≥ 7.8 mmol/L [140 mg/dL]).	531 women developed risk factors after 33 weeks, of whom 64 had LGA fetuses
Choi, 2020^(27)^	USA	Retrospective case-control study; Conference abstract	Fetal AC > 95th percentile, EFW > 90th percentile, mild to moderate polyhydramnios (AFI 25–35 cm) on two different ultrasounds, and/or severe polyhydramnios (AFI > 35 cm) on scan in the third trimester	100-g OGTT; Specific criteria not detailed	104 women in the third trimester with abnormal ultrasound findings; specific number of LGA fetuses is not reported
De Wit, 2019^(28)^	Netherlands	Retrospective cohort; Full-length article	Suspected accelerated fetal growth or macrosomia (clinical or ultrasound; fetal AC and/or EFW > 90th percentile), polyhydramnios, or maternal symptoms (rapid weight gain, polydipsia, excessive thirst)	75-g OGTT; WHO 1999 criteria**	183 women who underwent a second OGTT (median 33 weeks), of whom 150 had LGA fetuses
Fonseca, 2019^(29)^	Portugal	Prospective cohort; Full-length article	Universal rescreening invited between 32 and 36 weeks of pregnancy	75-g OGTT; IADPSG criteria***	334 women who completed the third trimester OGTT between 32 and 36 weeks, of whom 5 had LGA fetuses
Geifman, 2010^(30)^	Israel	Prospective cohort; Full-length article	Suspected macrosomia (EFW > 90th percentile) on scan at 37–40 weeks	100-g OGTT; Impaired glucose metabolism (treated as GDM) was diagnosed if there were ≥ 2 abnormal values according to Carpenter-Coustan criteria* OR 1 abnormal value according to NDDG criteria****	170 low-risk pregnant women who underwent OGTT at term (37–40 weeks), all of whom had LGA fetuses
Jasim, 2023^(31)^	Iraq	Prospective cohort; Full-length article	Suspected LGA (EFW > 90th percentile) on scan at 32 weeks	100-g OGTT; Carpenter-Coustan criteria*	153 pregnant women who underwent OGTT during the third trimester, all of whom had LGA fetuses
Maksym, 2016^(32)^	UK	Prospective cohort; Conference abstract	Suspected LGA (not specified), increased fetal AC, and/or polyhydramnios on a routine third trimester scan after 35 weeks	OGTT; Specific glucose load and diagnostic criteria are not detailed	77 women who underwent OGTT after 35 weeks; specific number of LGA fetuses is not reported
Mejia, 2025^(33)^	Spain	Retrospective cohort; Full-length article	Suspected fetal macrosomia (customized EFW ≥ 97th percentile) identified during a routine third-trimester scan between 34+0 and 36+6 weeks	100-g OGTT; NDDG criteria****	147 women who underwent OGTT; all of whom had suspected macrosomia
Sasson, 2019^(34)^	Israel	Retrospective cohort; Full-length article	Suspected LGA (EFW > 90th percentile) or polyhydramnios (AFI > 250 mm) at term (≥ 37 weeks of gestation)	100-g OGTT; Carpenter-Coustan criteria*	1,131 women with singleton pregnancies who completed a fasting OGTT at term; specific number of LGA fetuses is not reported
Shindo, 2021^(35)^	Japan	Retrospective cohort; Full-length article	LGA (EFW > 90th percentile), suspected macrosomia, polyhydramnios, or positive urine sugar levels) after 29 weeks of gestation	75-g OGTT; IADPSG criteria***	238 women with singleton pregnancies and no previous history of cesarean delivery who underwent an OGTT after 29 weeks of gestation; specific number of LGA fetuses is not reported
Sohn, 2020^(36)^	South Korea	Retrospective cohort; Full-length article	Suspected LGA (EFW > 90th percentile) on ultrasound in the third trimester	100-g OGTT; Carpenter-Coustan criteria*	169 singleton pregnant women who underwent a repeat OGTT in the third trimester; all of whom had LGA fetuses
Yekel, 2011^(37)^	Israel	Retrospective cohort; Conference abstract	Suspected macrosomia (not specified) or polyhydramnios in late pregnancy, as well as classic risk factors (family history of DM, previous GDM)	100-g OGTT; The study evaluated three different criteria: NDDG****, Carpenter-Coustan*, and one abnormal value	975 women in late pregnancy (GA not specified); specific number of LGA fetuses is not reported
Zilberberg, 2012^(38)^	Israel	Case-control; Conference abstract	Suspected macrosomia (EFW ≥ 90th percentile) or polyhydramnios (AFI ≥ 97th percentile) after 36 weeks of gestation	100-g OGTT; Carpenter-Coustan criteria*	900 patients who underwent an OGTT after 36 weeks; specific number of LGA fetuses is not reported

AC = abdominal circumference; AFI = amniotic fluid index; BMI = body mass index; DM = diabetes mellitus; EFW = estimated fetal weight; GA = gestational age; GCT = glucose challenge test; GDM = gestational diabetes mellitus; IADPSG = International Association of Diabetes and Pregnancy Study Groups; LGA = large for gestational age; NDDG = National Diabetes Data Group; OGTT = oral glucose tolerance test; WHO = World Health Organization.

* GDM is diagnosed using the Carpenter-Coustan criteria when at least two values meet or exceed the following thresholds: fasting, 95 mg/dL; 1-hour, 180 mg/dL; 2-hour, 155 mg/dL; and 3-hour, 140 mg/dL.

** GDM is diagnosed using the 1999 WHO criteria when at least one value meets or exceeds the following thresholds: fasting, 126 mg/dL; or 2-hour, 140 mg/dL.

*** GDM is diagnosed using the IADPSG criteria when at least one value meets or exceeds the following thresholds: fasting, 92 mg/dL; 1-hour, 180 mg/dL; or 2-hour, 153 mg/dL.

**** GDM is diagnosed using the NDDG criteria when at least two values meet or exceed the following thresholds: fasting, 105 mg/dL; 1-hour, 190 mg/dL; 2-hour, 165 mg/dL; and 3-hour, 145 mg/dL.

The geographical distribution of the research highlighted a strong concentration in the Middle East, primarily driven by six studies conducted in Israel^(24,25,30,34,37,38)^ and one in Iraq.^(31)^ The remaining research was geographically diverse, comprising five European studies from the United Kingdom,^(26,32)^ the Netherlands,^(28)^ Portugal,^(29)^ and Spain;^(33)^ one study from the USA;^(27)^ and two studies from Asian countries, Japan^(35)^ and South Korea.^(36)^

Regarding study design, the review included a predominance of retrospective cohorts (n=9) and prospective cohorts (n=4), alongside two case-control studies. Four of these reports were identified as conference abstracts,^(27,32,37,38)^ which inherently provided more limited granular data concerning obstetrical and neonatal outcomes compared to the full-text articles.

Concerning the diagnostic approach for late-onset dysglycemia, the majority of the studies (n=10) utilized the 100-g OGTT for third-trimester screening, primarily applying the Carpenter-Coustan or National Diabetes Data Group (NDDG) diagnostic thresholds. Three studies employed the 75-g OGTT according to regional guidelines.^(28,29,35)^ Furthermore, one study diverged from traditional OGTT screening, evaluating late-onset dysglycemia exclusively through rigorous home capillary blood glucose monitoring,^(26)^ while one conference abstract did not explicitly specify the glucose load used for the retest.^(32)^

The timing of the assessment and the clinical sonographic triggers for rescreening varied across the studies, capturing different phases of late pregnancy. Several studies evaluated late testing strictly at or beyond term (from 37 weeks of gestation onwards) to primarily counsel on immediate delivery modes.^(25,30,34,38)^ In terms of clinical indications, a targeted subset of studies focused strictly on isolated fetal macrosomia or LGA, requiring an EFW > 90th or ≥ 97th percentile for inclusion.^(30,31,33,36)^ Meanwhile, the remaining studies evaluated mixed high-risk cohorts, where third-trimester rescreening was triggered not only by EFW but also by isolated polyhydramnios, accelerated AC growth, or a combination of sonographic findings and persistent glycosuria.

### Summary of results

A targeted analysis of studies evaluating third-trimester rescreening reveals a clinically significant diagnostic yield for late-onset GDM, though the rates vary considerably depending on cohort composition and the specific indications for testing. Evaluating the conversion rate from a normal mid-trimester screening to a positive third-trimester diagnosis requires distinguishing between cohorts composed entirely of isolated LGA pregnancies and mixed high-risk populations. In studies where the rescreened population consisted exclusively of women presenting with third-trimester LGA or suspected macrosomia, the diagnostic yield was consistent across several distinct geographic cohorts.^(30,31,33,36)^ The incidence of late-onset GDM and the specific perinatal outcomes extracted from these and broader high-risk LGA subsets are compiled in chart 2. In these strictly isolated populations, the incidence of newly diagnosed late GDM clustered tightly around 13% to 15%. Specifically, Mejia et al.^(33)^ diagnosed GDM in 14.9% (22/147) of women tested exclusively for suspected fetal macrosomia. Similarly, Jasim et al.^(31)^ and Sohn et al.^(36)^ reported late GDM diagnoses in 13.7% (21/153) and 13.6% (23/169) of their isolated LGA cohorts, respectively. Conversely, Geifman et al.^(30)^ reported a notably lower yield in their strictly isolated LGA cohort, diagnosing impaired glucose metabolism in only 2.9% (5/170) of the women.

**Chart 2:** Incidence of late-onset GDM and associated clinical outcomes in LGA cohorts

Study (Author, Year)	LGA sample size (n)	Late-Onset GDM n(%)	Perinatal outcomes
Abu Shqara, 2022^(24)^	126	31(24.6)	Perinatal outcomes not reported specifically for the isolated LGA subgroup.
Barak, 2021^(25)^	147	6(4)	Perinatal outcomes not reported specifically for the isolated suspected macrosomia subgroup.
De Wit, 2019^(28)^	150	34(22.6)	Perinatal outcomes not reported specifically for the isolated LGA subgroup.
Fonseca, 2019^(29)^	5	1(20)	Perinatal outcomes not reported specifically for the isolated LGA subgroup.
Geifman, 2010^(30)^	170	5(2.9)	Elective procedures accounted for a higher proportion of surgical deliveries in the late-onset GDM group (85.7% vs. 41.1%). There were no reported cases of shoulder dystocia, postpartum hemorrhage, or severe perineal tears in the abnormal glucose group, whereas the NGT group had isolated cases of mild shoulder dystocia and 3rd-degree laceration.
Jasim, 2023^(31)^	153	21(13.7)	The late-onset GDM group had higher 3rd-trimester HbA1c (5.9% vs. 5.1%, p<0.05) and a significantly higher CS rate (76.2% vs. 51.5%, p<0.05). However, there were no significant differences between GDM and NGT groups regarding actual birth weight, confirmed LGA at delivery, shoulder dystocia, neonatal hypoglycemia, or NICU admission.
Mejia, 2025^(33)^	147	22(14.9)	Perinatal outcomes not reported specifically for the late-onset GDM subgroup.
Sohn, 2020^(36)^	169	23(13.6)	The late-onset GDM group delivered significantly earlier (38.0 vs. 38.9 weeks, *p*=0.003) with a higher CS rate (73.9% vs. 49.3%, *p*=0.028). Despite interventions, there were no significant differences in GA-adjusted birth weight or the incidence of obstetrical and neonatal complications, including shoulder dystocia, preterm birth, NICU admission, and neonatal hypoglycemia.

CS = cesarean section; GA = gestational age; GDM = gestational diabetes mellitus; HbA1c = glycated hemoglobin; LGA = large for gestational age; n = number of patients; NGT = normal glucose tolerance; NICU = neonatal intensive care unit

Studies that evaluated broader high-risk populations, including indications like polyhydramnios, glycosuria, or previous pathological GCTs, provide critical comparative insights when extracting their specific LGA subgroups. In Abu Shqara et al.,^(24)^ the overall diagnostic yield for the entire mixed cohort was 22.8% (85/372). However, when isolating the 126 women whose indication was specifically suspected of LGA, the diagnostic yield was even higher, at 24.6% (31/126), underscoring LGA as a strong independent predictor. De Wit et al.^(28)^ observed a similarly high conversion rate, reporting a 23.4% (43/183) overall GDM diagnosis rate in their mixed high-risk cohort, which closely mirrored the 22.6% (34/150) yield found in their large LGA-specific subgroup. In contrast, Barak et al.^(25)^ observed much lower detection rates overall. Their total mixed cohort of 272 women yielded an abnormal OGTT in only 5.9% (16/272) of cases, and the isolated suspected macrosomia subgroup demonstrated an even lower yield of 4.0% (6/147). Furthermore, in a universal rescreening trial by Fonseca et al.,^(29)^ the overall diagnostic yield was 13.5% (45/334); however, the LGA subgroup in this cohort was too small (n=5) to draw robust conclusions, though one of those five (20%) was diagnosed with GDM.

Several other studies assessed late GDM in broad high-risk populations without providing granular diagnostic yields specifically for their LGA subsets. Nonetheless, these populations, which heavily featured LGA and polyhydramnios as primary screening triggers, demonstrated substantial late-GDM incidences. Large retrospective analyses reported yields of 13.2% (150/1131) in Israel and 18.2% (49/268) in Japan.^(34,35)^ Smaller prospective setups identified even higher conversion rates, such as 25.9% (27/104) in Choi et al.^(27)^ and 32.5% (25/77) in Maksym et al.^(32)^ Notably, Cauldwell et al.^(26)^ utilized an intensive 7-day home capillary blood glucose monitoring protocol rather than a standard OGTT for high-risk women, triggered predominantly by glycosuria and increased fetal AC. This rigorous methodology yielded a remarkably high diagnosis rate, identifying late-onset GDM in 46.0% (244/531) of the tested population. Despite the high yields demonstrated in most of the literature, Yekel et al.^(37)^ offered a dissenting perspective. In a large retrospective cohort of 975 women who underwent late OGTT after a normal mid-trimester GCT, the diagnostic yield ranged from only 2.7% to 5.6%, depending on the criteria applied (NDDG versus Carpenter-Coustan). The authors concluded that in the context of a previously normal screening, suspicion of macrosomia or polyhydramnios were actually poor predictors of late-onset GDM, arguing that classic maternal risk factors remained the true drivers of false-negative mid-trimester results.

When evaluating the impact of a late GDM diagnosis on obstetric and neonatal outcomes, it is also crucial to distinguish whether the reported data apply exclusively to pregnancies complicated by isolated LGA fetuses or to broader mixed high-risk cohorts. Regardless of the cohort composition, a late-onset GDM diagnosis definitively altered maternal obstetric management, consistently leading to increased medicalization. In studies evaluating strictly isolated LGA populations, women in the late-GDM group experienced significantly higher rates of cesarean sections (CS), with Jasim et al.^(31)^ reporting a CS rate of 76.2% versus 51.5% in the normal glucose tolerance (NGT) group (p<0.05), and Sohn et al.^(36)^ observing 73.9% versus 49.3% (p=0.028). Additionally, Geifman et al.^(30)^ highlighted that elective procedures accounted for 85.7% of all surgical deliveries in the late-onset GDM group, compared to only 41.1% in the NGT group.

Similarly, studies analyzing mixed high-risk cohorts corroborated this trend with robust adjusted odds ratios. Abu Shqara et al.^(24)^ reported increased odds of labor induction (OR 1.95, 95% CI 1.15–3.33) and elective CS (OR 2.21, 95% CI 1.17–4.20) among women newly diagnosed with GDM. Shindo et al.^(35)^ observed that late GDM more than doubled the odds of an overall CS (aOR 2.41, 95% CI 1.07–5.43) and more than tripled the odds of an elective CS (aOR 3.60, 95% CI 1.27–10.19). Zilberberg et al.^(38)^ and Sasson et al.^(34)^ echoed these findings, with the former noting a CS rate of 59% in the GDM group versus 25% in controls (p<0.01), and the latter reporting a more than twofold increase in the final CS rate (24.7% vs. 10.5%). Fonseca et al.^(29)^ also demonstrated that an abnormal third-trimester OGTT was an independent risk factor for cesarean delivery (aOR 2.57, 95% CI 1.14–5.81) across their cohort.

Despite this change in obstetric interventions, the extracted data did not demonstrate consistent differences in acute neonatal morbidities in several cohorts. Across targeted isolated LGA populations^(30,31,36)^ and some mixed high-risk cohorts,^(24,35)^ no statistically significant differences were observed between the late-onset GDM and NGT groups regarding the incidence of shoulder dystocia, severe perineal tears, postpartum hemorrhage, or neonatal intensive care unit (NICU) admissions. Even after adjusting for earlier gestational ages at delivery (38.0 vs. 38.9 weeks, p=0.003), actual birth weights remained comparable between the GDM and NGT groups in the study conducted by Sohn et al. (3,512 g vs. 3,603).^(36)^ However, some mixed cohorts did detect elevated neonatal risks among the late-diagnosed group. Sasson et al.^(34)^ reported higher rates of neonatal hypoglycemia (14.7% vs. 11.5%, p=0.001), and Zilberberg et al.^(38)^ noted a significantly higher rate of a composite adverse neonatal outcome (13% vs. 4%, p=0.02) along with longer neonatal hospitalizations (3.5 vs 2.8 days, p=0.016).

Importantly, these perinatal outcomes must be interpreted through the lens of active clinical intervention. In all included cohorts, women who received a late diagnosis of GDM were subsequently submitted to standard-of-care disease management, which included medical nutrition therapy, glycemic monitoring, and occasionally pharmacotherapy. Consequently, the lack of significant differences in perinatal complications in many studies likely reflects the success of actively treated pregnancies, potentially masking the true complication rates of untreated late-onset dysglycemia. The protective effect of this active management is powerfully illustrated by Cauldwell et al.,^(26)^ who observed that high-risk women who tested negative for late-onset GDM, and were thus returned to routine antenatal care without dietary or glycemic intervention, actually exhibited significantly higher rates of confirmed birth macrosomia (25.4% vs. 16.0%, p<0.001) compared to women diagnosed and actively treated for late GDM. This strongly suggests that the diagnostic yield and subsequent treatment of late-onset GDM effectively curtail excessive fetal growth and its associated complications.

## Discussion

The central question driving this scoping review cannot be definitively answered with the current evidence. While identifying late-onset dysglycemia yields diagnoses in 2.9% to 24.6% of cases, the primary barrier to a definitive clinical consensus is the complete absence of randomized controlled trials (RCTs). Without RCTs randomizing women with third-trimester LGA fetuses to routine care versus re-screening, it is impossible to determine if late diagnosis significantly improves neonatal outcomes or merely drives iatrogenic interventions. Currently, identifying late GDM does not consistently translate into statistical reductions in severe morbidities, like shoulder dystocia. However, this represents a treatment paradox: diagnosed patients ethically receive standard-of-care management, which mitigates overgrowth but masks the natural history of the disease. Consequently, the late GDM label carries a significant iatrogenic burden, frequently driving anxiety-induced increases in labor inductions and elective cesarean deliveries, often without the actual birthweight justifying the surgical delivery.

A major challenge in translating the current evidence into practice is the profound methodological heterogeneity across studies and the unaddressed systemic costs. The extracted data stem from fundamentally different study designs analyzing varying populations (isolated LGA versus mixed high-risk cohorts), drastically different gestational ages at the time of testing (29 to 40 weeks), and conflicting diagnostic criteria (75-g versus 100-g OGTTs, or even home capillary monitoring). This lack of standardization precludes the establishment of a universal screening protocol. Furthermore, the financial and systemic impacts of routine third-trimester rescreening remain entirely unknown. The cascade of increased medicalization, encompassing widespread OGTTs, specialized medical consultations, and higher surgical delivery rates, must be carefully weighed against the potential cost savings of avoiding macrosomia-related morbidities through comprehensive cost-effectiveness analyses before any universal recommendation can be made. This clinical scenario is further complicated by diagnostic gaps in early pregnancy. For instance, institutional data shows that 28% of mothers with macrosomic infants met sensitive GDM criteria early in gestation but went unrecognized until delivery,^(9)^ reinforcing that screening inaccuracies earlier in pregnancy can confound third-trimester management dilemmas.

Subjecting all patients with LGA fetuses to a repeat OGTT may also be clinically inefficient, as a persistent clinical dogma erroneously assumes that excessive fetal growth inherently conceals undetected glucose intolerance. Emerging evidence highlights that fetal macrosomia is multifactorial and heavily influenced by glucose-independent pathways. For instance, maternal third-trimester lipid profiles, specifically isolated hypertriglyceridemia, independently correlate with increased fetal weight even in strictly normoglycemic pregnancies.^(39,40)^ Furthermore, pre-pregnancy obesity and excessive gestational weight gain are also established primary drivers. Research indicates that mothers of LGA infants often exhibit normal beta-cell function and metabolic profiles identical to healthy mothers, completely diverging from the physiological dysfunction seen in true GDM.^(41,42)^

Rather than relying on global estimated fetal weight, the literature suggests that fetal growth asymmetry is a vastly superior clinical predictor of late dysglycemia. Fetuses exposed exclusively to late-pregnancy hyperglycemia exhibit a paradoxical growth pattern characterized by an accelerated, isolated increase in AC rather than symmetric global macrosomia.^(43)^ Consequently, an AC at or above the 95th percentile may serve as a stronger independent trigger for glucose re-screening.^(26,44)^ Identifying this specific subset of patients is crucial, as untreated third-trimester hyperglycemia carries severe metabolic consequences, including increased childhood adiposity and a four-fold higher risk of maternal postpartum type 2 diabetes.^(45)^ When correctly detected and controlled, late-onset hyperglycemia management yields quantifiable reductions in CS, preterm births, and LGA infants, underscoring the therapeutic value of identifying the appropriate patients.^(46)^

The robustness of this scoping review is anchored in its systematic methodology and strict adherence to PRISMA-ScR guidelines, which allowed for a comprehensive mapping of this specific clinical dilemma. However, the findings must be interpreted in light of the primary studies’ limitations, notably the aforementioned methodological heterogeneity and a marked geographical bias, with a heavy concentration of Middle Eastern cohorts limiting global generalizability. The predominance of retrospective designs also introduces selection bias, as clinicians may have selectively tested patients with the most severe ultrasonographic presentations. Ultimately, while third-trimester GDM rescreening can identify genuine metabolic dysfunction with significant long-term implications, the current lack of RCTs, standardized protocols, and economic evaluations makes it premature to recommend universal re-testing solely based on an isolated LGA finding.

## Conclusion

The current body of evidence remains limited, and there is a lack of robust studies that definitively support or refute the hypothesis that late-onset GDM is the primary driver of excessive growth in previously screen-negative patients presenting with an isolated LGA fetus. Consequently, routine and indiscriminate repeat glucose testing in the third trimester cannot be universally recommended. Instead, each case must be evaluated carefully on an individual basis, weighing the presence of additional GDM risk factors and adhering to local institutional protocols. Moving forward, ethically designed interventional trials are urgently needed to clarify the true clinical value of late re-screening and targeted treatment in this specific population. In the interim, obstetric care providers must remember that maternal glycemic alterations are not the sole etiology of an LGA fetus, and a broader clinical perspective encompassing maternal weight, genetics, and lipid metabolism is essential for optimal care.

## Data Availability

The research data are described in the article presented.
