# Supplementary material for: Should glucose screening be repeated for large-for-gestational-age fetuses in the third trimester of non-diabetic pregnancies? A scoping review
Source: Rev Bras Ginecol Obstet. 2026 Oct 30;48:e-rbgo76. doi: 10.61622/rbgo/2026rbgo76 (PMC13754073; doi:10.61622/rbgo/2026rbgo76)
Supplement: Supplementary file 1 [file 1806-9339-rbgo-48-e-rbgo76-Suppl01.pdf]

# Supplementary material

**Table 1S.** Search strategy

Comprehensive systematic electronic searches were conducted on June 25, 2026, across three main databases to identify relevant literature without chronological restrictions.

| |
|---|
| In PubMed (MEDLINE) via the National Center for Biotechnology Information (NCBI) platform, the search string utilized combined Medical Subject Headings (MeSH) and text-word terms:<br><br>((“Fetal Macrosomia”[Mesh] OR “Large for Gestational Age”[tiab] OR “LGA”[tiab] OR “macrosomia”[tiab] OR “macrosomic”[tiab]) AND (“Pregnancy Trimester, Third”[Mesh] OR “third trimester”[tiab] OR “late pregnancy”[tiab]) AND (“Mass Screening”[Mesh] OR “Glucose Tolerance Test”[Mesh] OR “screening”[tiab] OR “rescreening”[tiab] OR “re-screening”[tiab] OR “repeat test*”[tiab] OR “re-test*”[tiab] OR “retest*”[tiab] OR “OGTT”[tiab] OR “glucose screening”[tiab]) AND (“Diabetes, Gestational”[Mesh] OR “gestational diabetes”[tiab] OR “GDM”[tiab])), yielding 113 records.<br><br>In Embase via the Elsevier platform, the strategy integrated Emtree structures and free-text titles and abstracts:<br><br>((‘macrosomia’/exp OR ‘large for gestational age’/exp OR ‘large for gestational age’:ti,ab OR ‘lga’:ti,ab OR ‘macrosomia’:ti,ab OR ‘macrosomic’:ti,ab) AND (‘third trimester pregnancy’/exp OR ‘third trimester’:ti,ab OR ‘late pregnancy’:ti,ab) AND (‘screening’/exp OR ‘glucose tolerance test’/exp OR ‘screening’:ti,ab OR ‘rescreening’:ti,ab OR ‘re-screening’:ti,ab OR ‘repeat test*’:ti,ab OR ‘re-test*’:ti,ab OR ‘retest*’:ti,ab OR ‘ogtt’:ti,ab OR ‘glucose screening’:ti,ab) AND (‘gestational diabetes mellitus’/exp OR ‘gestational diabetes’:ti,ab OR ‘gdm’:ti,ab)), retrieving 380 records.<br><br>In SciELO via its native integrated search interface, a multilingual trilingual approach (English, Portuguese, and Spanish) was deployed to capture regional literature:<br><br>((“large-for-gestational-age” OR “LGA” OR “macrosomia” OR “macrosomia fetal” OR “feto grande para a idade gestacional” OR “gigante para edad gestacional”) AND (“third trimester” OR “terceiro trimestre” OR “tercer trimestre”) AND (“screening” OR “re-screening” OR “rescreening” OR “repeat testing” OR “reteste” OR “repetição” OR “tamizaje” OR “ogtt” OR “totg”) AND (“gestational diabetes” OR “GDM” OR “diabetes gestacional”)), returning 1 record. |

No automation filters or language limits were hardcoded into the initial search strings to prevent database truncation errors. Instead, all geographic and language eligibility criteria were manually enforced during the Rayyan screening phase.

**Table 2S.** Data extraction instrument

| |
|---|
| A customized data extraction form was developed and implemented within the Rayyan platform to capture study characteristics and specific clinical outcomes. Since standard JBI data extraction templates do not inherently include specific diagnostic fields for late-onset gestational diabetes and fetal growth metrics, the following standardized data points were extracted from all eligible studies:<br>• Study demographics and design: Author, year of publication, country of origin, and study design.<br>Clinical baseline and selection: Gestational age at critical checkpoints, and study inclusion criteria.<br>Screening parameters and criteria: Type of GDM test used, and specific GDM definitions/screening protocols applied.<br>• Cohort data: Total number of participants, and total number of LGA fetuses.<br>• Retesting and diagnostic yield: Number of LGA cases with a normal second-trimester screening that subsequently developed third-trimester GDM, and the absolute number of new GDM cases.<br>Outcomes: Core findings regarding maternal and neonatal management and overall diagnostic yield. |
